# Measuring Cerebrovascular Reactivity: Sixteen Avoidable Pitfalls

**DOI:** 10.3389/fphys.2021.665049

**Published:** 2021-07-07

**Authors:** Olivia Sobczyk, Jorn Fierstra, Lakshmikumar Venkatraghavan, Julien Poublanc, James Duffin, Joseph A. Fisher, David J. Mikulis

**Affiliations:** ^1^Department of Anaesthesia and Pain Management, University Health Network, University of Toronto, Toronto, ON, Canada; ^2^Joint Department of Medical Imaging and the Functional Neuroimaging Laboratory, University Health Network, Toronto, ON, Canada; ^3^Department of Neurosurgery, University Hospital Zurich, Zürich, Switzerland; ^4^Department of Physiology, University of Toronto, Toronto, ON, Canada; ^5^Institute of Medical Science, University of Toronto, Toronto, ON, Canada

**Keywords:** carbon dioxide, cerebral blood flow, vascular responses, cerebrovascular reactivity, cerebrovascular reactivity to carbon dioxide

## Abstract

An increase in arterial PCO_2_ is the most common stressor used to increase cerebral blood flow for assessing cerebral vascular reactivity (CVR). That CO_2_ is readily obtained, inexpensive, easy to administer, and safe to inhale belies the difficulties in extracting scientifically and clinically relevant information from the resulting flow responses. Over the past two decades, we have studied more than 2,000 individuals, most with cervical and cerebral vascular pathology using CO_2_ as the vasoactive agent and blood oxygen-level-dependent magnetic resonance imaging signal as the flow surrogate. The ability to deliver different forms of precise hypercapnic stimuli enabled systematic exploration of the blood flow-related signal changes. We learned the effect on CVR of particular aspects of the stimulus such as the arterial partial pressure of oxygen, the baseline PCO_2_, and the magnitude, rate, and pattern of its change. Similarly, we learned to interpret aspects of the flow response such as its magnitude, and the speed and direction of change. Finally, we were able to test whether the response falls into a normal range. Here, we present a review of our accumulated insight as 16 “lessons learned.” We hope many of these insights are sufficiently general to apply to a range of types of CO_2_-based vasoactive stimuli and perfusion metrics used for CVR.

## Introduction

For the last two decades, our laboratory has been engaged in interrogating cerebral vascular function. The overarching approach has been to observe changes in regional brain flow in response to a vasoactive stimulus. This is referred to as cerebrovascular reactivity, or cerebral vascular reactivity (CVR). There are a variety of well-described vasoactive stimuli and outcome measures. Our laboratory employs precise targeting of end-tidal PCO_2_ (P_ET_CO_2_) and blood oxygen-level-dependent (BOLD) magnetic resonance imaging (MRI) as the surrogate measure of cerebral blood flow (CBF). The ability to precisely duplicate a stimulus has enabled us to, retrospectively and prospectively, re-examine the way we generate and interpret our data. Indeed, over time we have identified unwarranted assumptions—including some in which we had high levels of confidence that led to weak methodology and misguided data analysis, and conclusions, regrettably, some after they appeared in our own published work. We have published most of these insights in multiple separate papers. Nevertheless, we thought that it would be useful to the CVR community for us to review these insights in summary format in one paper. We believe the principles can selectively inform on the strengths and limitations of other CVR studies performed under a range of stimuli such as infusion of acetazolamide, breath hold, inspiration of fixed inspired PCO_2_ (FICO_2_), and with the use of outcome measures such as transcranial Doppler (TCD) and various MRI methods.

### Stimulus Response

Cerebral vascular reactivity is a provocative cerebral vascular test analogous to a cardiac stress test. For both, provocation is required to elicit a flow response exceeding baseline perfusion to ascertain the flow reserve. In the case of the cardiac stress testing, treadmill exercise or vasoactive agent stressors indicate the presence of flow deficits in the form of chest pain, ECG changes, or flow reductions in cardiac perfusion imaging. The results are then interpreted using angiographic findings of the vascular patho-anatomy.

#### Stressors

For studies of the heart, a standard stimulus may consist of aerobic exercise pushed to the threshold where anaerobic metabolism becomes active. When using pharmacological vasoactive agents such as adenosine, regadenoson, and dipyridamole as standard stimuli, uniformity of the vasodilatory stimulus is achieved by supramaximal dosing, that is, the dose beyond which no further flow response occurs.

In the brain, activation of neurovascular coupling throughout the brain is not an option as there is no safe stimulus that can activate all neurons. However, global vasoactive stimulation can be affected pharmacologically by the intravenous injection of hypotensive agents or using carbonic anhydrase blockers such as acetazolamide. The former is not considered safe for this indication. Acetazolamide can be injected at supramaximal response doses, but its time course is not predictable (Dahl et al., 1995; Grossmann and Koeberle, 2000) and results in frequent uncomfortable side effects (Ringelstein et al., 1992; Dahl et al., 1995; Grossmann and Koeberle, 2000; Saito et al., 2011).

Hypercapnia, defined an increased arterial partial pressure of CO_2_ (PaCO_2_), is easily implemented, safe, well-tolerated, and is therefore, the most used stressor. Each mmHg increase in PaCO_2_ increases CBF by ~6–8% (Kety and Schmidt, 1948; Willie et al., 2012; Al-Khazraji et al., 2019). Supramaximal levels of PaCO_2_ (greater than 90 mmHg (Reivich, 1964) cannot be used to obtain a standard stimulus as levels that exceed 50–60 mmHg are very uncomfortable (Willie et al., 2012) and levels acutely exceeding ~80 mmHg begin to cause confusion and unconsciousness. However, implementation of a known stimulus requires the ability to precisely target PaCO_2_. This is not a trivial task. First, while it may seem that PaCO_2_ should simply be a function of the inspired fractional concentration of CO_2_ (FICO_2_), it is in fact also a function of the minute ventilation, which itself changes when inhaling CO_2_ (Fisher, 2016). Since the change in minute ventilation in response to a change in FICO_2_ cannot be predicted, *a particular PaCO_2_ cannot be targeted* by designating the FICO_2_ (Peebles et al., 2007).

Measuring PaCO_2_, the independent variable of CBF, is problematic. The only non-invasive measure of PaCO_2_ available is the P_ET_CO_2_, but, unfortunately, without rebreathing, it is not a reliable surrogate for PaCO_2_ (Jones et al., 1972). The P_ET_CO_2_ does approach PaCO_2_ only with complete rebreathing (Duffin and McAvoy, 1988), and prospective targeting using sequential gas delivery (SGD; Ito et al., 2008; Fisher et al., 2016). End-tidal forcing (Robbins et al., 1982) is able to target P_ET_CO_2_, but its equivalence to PaCO_2_ has not been demonstrated (Jones et al., 1972; McDonald et al., 2002; Tymko et al., 2016). When using breath hold as the vasoactive stimulus, the P_ET_CO_2_ is related to breath hold duration, but PaCO_2_ is unknown (Totaro et al., 1999).

#### Stress Indicators

The stress indicator, CBF, is measured indirectly by surrogate measures. All surrogates can be characterized by fidelity to flow and their limitations in this regard. TCD measures velocity with high temporal resolution. The output, however, reflects velocity only in a single arterial segment rather than the brain parenchyma. Even so, its relation to flow assumes no particular corresponding change in vessel diameter with the stimulus, which may not hold (Willie et al., 2012; Verbree et al., 2014; Al-Khazraji et al., 2019). In our laboratory, we use BOLD signal changes as surrogates for changes in flow. These signals have high temporal resolution and high spatial resolution, providing whole brain maps of tissue perfusion. Although BOLD reflects flow-induced deoxyhemoglobin dilution, to its credit the signal has a reasonably linear relation to flow at moderate levels of hypercapnia (Hoge et al., 1999). Both TCD and BOLD are internally consistent but are poorly correlated (Burley et al., 2020). Arterial spin labeling uses magnetic labeling of water protons as a flow tracer and is a very good measure of flow. However, its limitations for CVR include imperfect labeling efficiency that can change from patient to patient based on difference in anatomy, and the magnetic label decays as a function of T1-relaxation over time. Delays in arrival time caused by steno-occlusive disease can result in problems with labeled proton localization and increasing image noise.

## CVR Measures

### Basic CVR Measures

Failing precise targeting of PaCO_2_, a reasonable fallback position is to assume that the ΔP_ET_CO_2_ is close to ΔPaCO_2_ and therefore can be used to index the change in flow for the stimulus by dividing it by ΔP_ET_CO_2_ and defining the stress indicator as the slope of line of best fit, CVR (Vesely et al., 2001; Sobczyk et al., 2014).

We initially considered the magnitude of the CVR as reflecting the vasodilatory vigor of the underlying vasculature (Bhogal et al., 2014). In patients with known vascular pathology, we identified specific regions of interest (ROI) in some patients where, rather than flow increasing in response to a stressor, it declined. Such areas of “steal” in response to hypercapnia had been described more than 40 years ago (Brawley, 1968; Symon, 1969) and have been reviewed recently (Fisher et al., 2018).

### Enhanced CVR Model and Physiological Interpretations

We also developed a more comprehensive model to explain additional behaviors of the vasculature during hypercapnia. This model consisted of blood vessels organized in a series of hierarchical vascular beds where each downstream bed has a greater flow potential than its supply vessels (Duffin et al., 2017, 2018). Consequently, on the application of a vasoactive stimulus, vascular territories perfused in parallel from a common source must compete for inflow such that increased inflow to beds capable of more robust vasodilation is at the expense of those with less robust vasodilation, i.e., with steal (Sobczyk et al., 2014). The presence of steal is known to be a strong marker for risk of stroke (Reinhard et al., 2014) and is therefore important to identify. Importantly, the absence of steal indicates sufficient collateral blood flow to meet the flow requirements downstream from the stenosis and seems protective for stroke (Bang et al., 2008; Sheth et al., 2016; Tan et al., 2016).

This view is the basic model we followed in studying CVR in the first 434 patients examined (Spano et al., 2013). While maintaining consistency in the methodology of our studies, we nevertheless explored alternatives to our initial underlying assumptions. The aim of the remainder of this paper assembles some of the subtle, but retrospectively obvious lessons we learned over the last two decades about optimizing the stressor, developing new stress indicators, and furthering the understanding of cerebral vascular physiology.

#### The Two-Point Stimulus

As defined above, the vasoactive stimulus is the PaCO_2_. Note that when the hypercapnic stressor is implemented by breath hold or fixing the FICO_2_, the magnitude of the stressor, that is, the change in the independent variable PaCO_2_, is unknown. With breath hold, ΔCBF is a function of time and can only be indexed by breath hold duration, which correlates poorly with ΔPaCO_2_. For FICO_2_ inhalation, ΔP_ET_CO_2_ is known, but not ΔPaCO_2_. As such, CVR can only have a binary outcome: either positive or negative (steal absent or present). This information is retained despite the uncertainty in ΔPaCO_2_ because the denominator ΔP_ET_CO_2_ is always positive, leaving the sign of the slope to depend only on the numerator, ΔCBF.

### CVR Beyond the Two-Point Stimulus

To obtain information beyond this binary labeling, we set out to address one major unknown by precisely targeting the PaCO_2_. For this purpose, we developed a system involving SGD (Slessarev et al., 2007; Fisher et al., 2016). SGD enables the establishment of P_ET_CO_2_ within 2 mmHg of a target, independent of the level and pattern of breathing. A further benefit of SGD is that the difference between P_ET_CO_2_ and PaCO_2_ falls within the range of error of the blood gas analyzer, making the equivalent value to PaCO_2_ accessible non-invasively (Ito et al., 2008; Fierstra et al., 2011, 2013). Also employing SGD, Willie et al. (2012) reported a regression of PaCO_2_ vs. P_ET_CO_2_ over a range of 15–65 mmHg had slope 0.88 with a *r*^2^ of 0.98 and Bland–Altman analysis showing a bias where P_ET_CO_2_ exceeded PaCO_2_ by about 5 mmHg at the highest PCO_2_ levels.

Note that henceforth in this paper, when referring to a stressor administered *via* SGD, we will cite PaCO_2_ when referring to the stimulus and P_ET_CO_2_ when referring to the measured parameter.

#### CVR in Health

In healthy people the relationship between PaCO_2_ and CBF is sigmoidal with a midpoint close to baseline PaCO_2_ showing vasodilatory and vasoconstrictor reserves (Battisti-Charbonney et al., 2011; Bhogal et al., 2014; Duffin et al., 2017). The sigmoid response characteristics vary between white matter and gray matter (Bhogal et al., 2015) and indeed between more specific anatomical locations (Sobczyk et al., 2015; van Niftrik et al., 2017; Duffin et al., 2018). Nevertheless, in health, the slope of a voxel-wise line of best fit between P_ET_CO_2_ and CBF provides a reasonably accurate, simplifying, linear representation of the sigmoidal CO_2_-CBF response (Bhogal et al., 2014; Sobczyk et al., 2014). Sobczyk et al. (2015) and McKetton et al. (2018) have provided an atlas mapping normative CVR with voxel-wise mean ± SD for CVR in health.

#### CVR in Neurovascular Disease

##### The Shape of the PCO_2_–Flow Relationship

**Figure 1:**
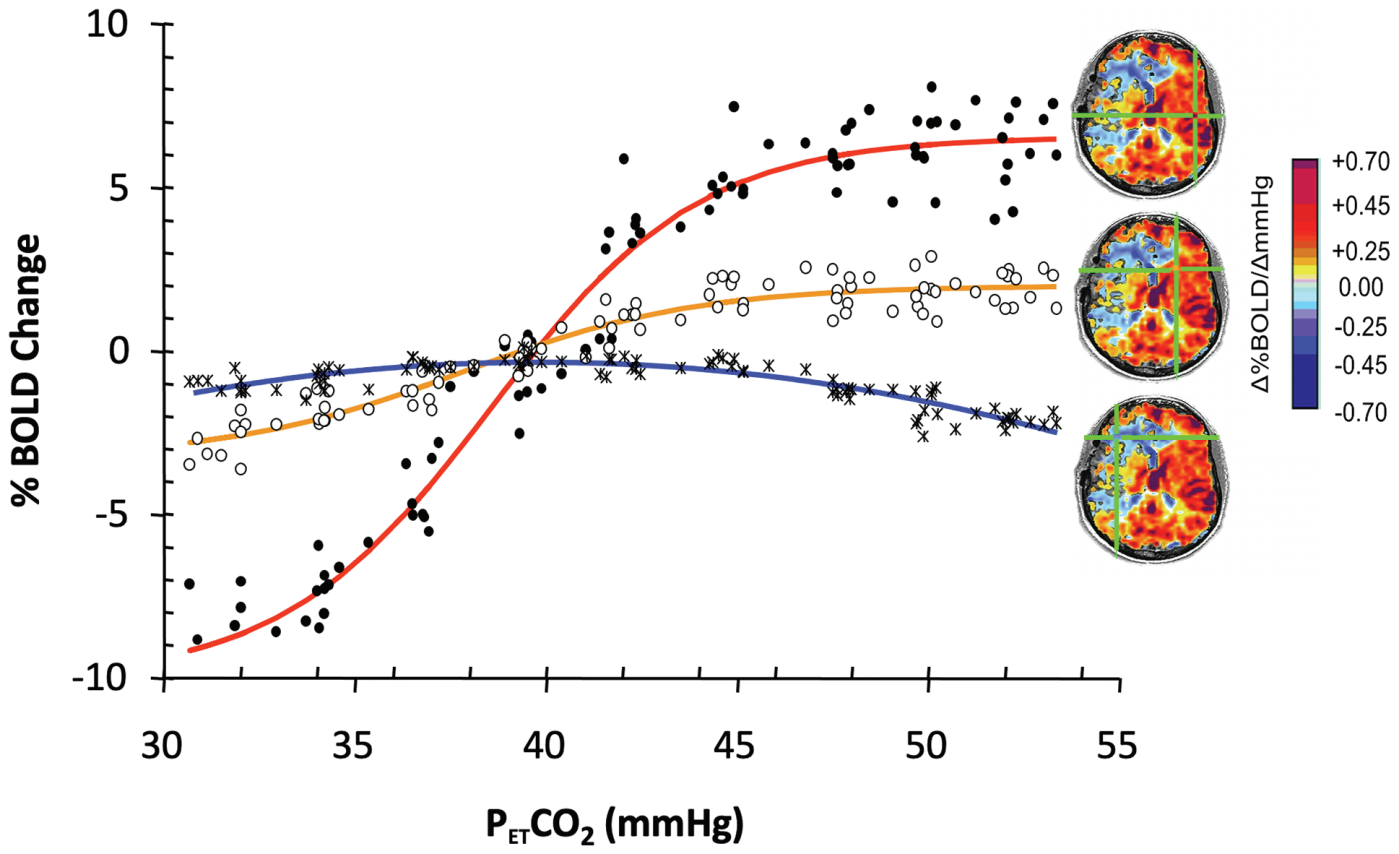
The effect of progressive weakening of vascular response to PaCO_2_ on the calculation of cerebral vascular reactivity (CVR). Data show blood oxygen-level-dependent (BOLD) responses to a range of P_ET_CO_2_ in selected ROI in an 18-year-old male with moyamoya disease affecting predominantly the right MCA territory. With progressively weaker vascular responsiveness, the sigmoid relationship (red curve from healthy cortex) weakens, the slope flattens (yellow curve, from moderately compromised territory), and finally, flow decreases instead of increases (steal: blue curve, severely compromised territory) in favor of the more robust vascular beds. Overall fitting a straight line to the data between P_ET_CO_2_ 40 and 50 mmHg results in a good fit for the red and yellow lines, less so for the biphasic blue line with slope depending on baseline and the highest PaCO_2_. Modified from Sobczyk et al. (2014).

In the presence of neurovascular disease, the sigmoidal relationship of flow to PaCO_2_ is degraded such that curves become flattened (Harper and Glass, 1965; Ringelstein et al., 1988; Sobczyk et al., 2014). Figure 1 represents flow in selected voxels over a range of PaCO_2_ in a patient with cerebrovascular disease.

**Figure 2:**
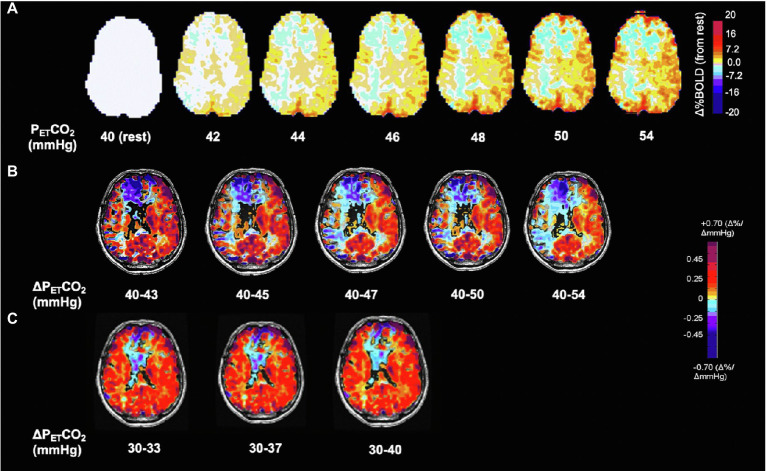
Voxel-wise changes of BOLD signal and CVR as a function of ΔP_ET_CO_2_ in the hypocapnic and hypercapnic range. Same subject as Figure 1. **(A)** BOLD signal change in % from baseline P_ET_CO_2_ 40 mmHg shown in interval increases of 2 mmHg. Note progressive changes in BOLD signal at every increment of P_ET_CO_2_ of 2 mmHg. **(B)** Effect of CVR calculated as ΔBOLD/ΔP_ET_CO_2_ on differences in ΔP_ET_CO_2_. Note increases in the extent of steal with greater changes in PaCO_2_ demonstrating its shortcomings for normalization for ΔP_ET_CO_2_. Standardization therefore results from using a reproducible stimulus. **(C)** CVR in the hypocapnic range is radically different from the hypercapnic range (see also Figure 3). Respective color scales on right.

Note that a suitably strong stressor is required to stimulate a sufficiently robust vasodilation in healthier vascular territories such that blood flow is preferentially directed to those territories and away from those with a weaker vasodilatory response, resulting in reduced flow or steal. The greater the vasodilation in the healthy vasculature, the greater the sensitivity for exposure of vasodilatory reserve (Figure 2).

**Figure 3:**
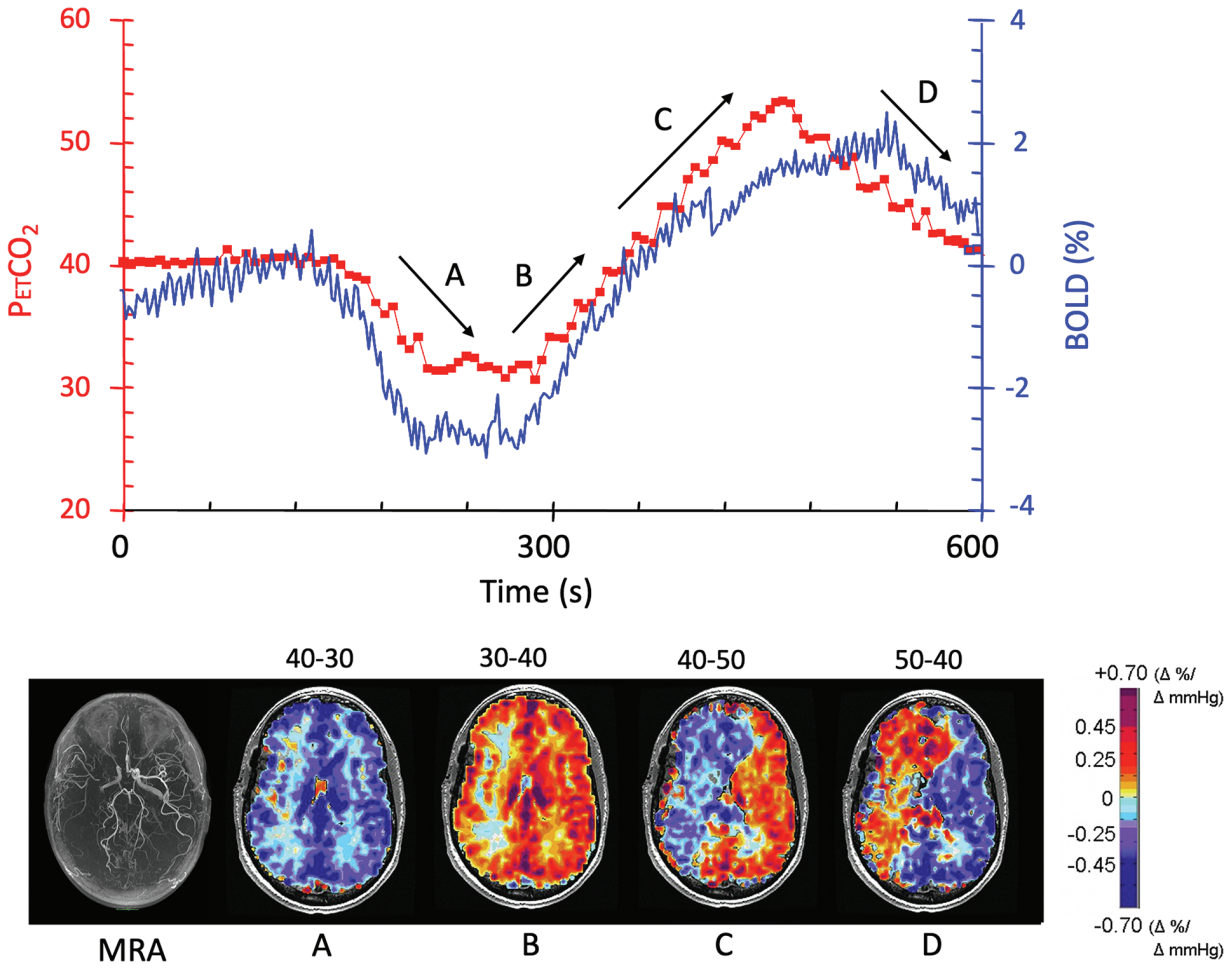
The effect of direction of change of PaCO_2_ on CVR. The patient is the same individual whose data are shown in Figures 1, 2. The experimental paradigm of changes in P_ET_CO_2_ is shown in red in the graph; BOLD signal changes are shown in blue. CVR maps shown in the lower figure correspond to the periods depicted on the graph, with arrows showing the direction of change in P_ET_CO_2_. **(A)** As P_ET_CO_2_ is reduced from 40 mmHg to 30 mmHg, there is a symmetrical reduction in BOLD signal in the white and gray matter in both hemispheres. **(B)** The mirror image change in P_ET_CO_2_ from 30 mmHg to 40 mmHg results in an asymmetrical change where flow increases in the left hemisphere while decreasing in the right (steal). **(C)** Continuation of increase in P_ET_CO_2_ to 50 mmHg exacerbates the flow discrepancies. **(D)** Reducing the P_ET_CO_2_ from 50 mmHg to 40 mmHg reduces flow in the healthier left hemisphere as expected when P_ET_CO_2_ falls. However, the blood vessels in right hemisphere paradoxically increase their flow, a feature attributed to the improved perfusion pressure as left hemisphere vessels constrict. Modified from Sobczyk et al. (2014).

From these considerations, we have the first four lessons:

1. When CBF responses to changes in P_ET_CO_2_ are curved, a two-point stressor will result in a reduced CVR.
2. The less sigmoidal the flow vs. PaCO_2_ curve, the more tenuous the connection between the CVR calculation and the vascular reactivity and the more the CVR is affected by the initial PaCO_2_ and ΔPaCO_2_ (see Figure 2).
3. Small differences in the ΔPaCO_2_ (~2 mmHg) result in measurable changes in CVR throughout the hypocapnic and hypercapnic ranges (Figure 2).
4. The CVR depends on whether the ΔPaCO_2_ is applied in the hypocapnic or hypercapnic range and the direction of change (see also Ide et al., 2003; Coverdale et al., 2014; Figure 3).

## Aspects of the Stimulus Affecting CVR

### Baseline PCO_2_ and CVR

Following from (2): What baseline PCO_2_ should be used for measuring CVR? In the first decade, we assumed that the normal PCO_2_ for humans was nominally 40 mmHg and would provide a universal starting point. However, over the years we found that resting P_ET_CO_2_ varied between 30 mmHg and over 47 mmHg, but each baseline P_ET_CO_2_ remained mostly constant over time, in some people over the many years in which we followed them.

**Figure 4:**
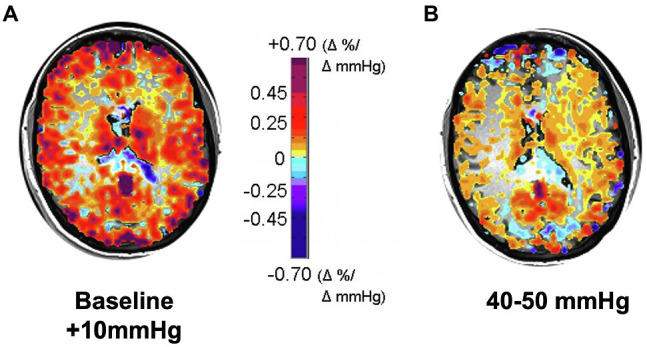
CVR responses to two 10 mmHg increases in P_ET_CO_2_: **(A)** from subject’s actual baseline to baseline + 10 mmHg, showing higher CVR; **(B)** from 40 to 50 mmHg, showing that CVR is globally reduced. Subject was a healthy active 30-year-old female with resting P_ET_CO_2_ ranging between 28 and 30 mmHg on different days, verified by a direct measure of PaCO_2_; her commensurately reduced bicarbonate level indicated that this was a longstanding condition, not due to acute hyperventilation.

The alternate approach we considered was that of identifying a participant’s resting PCO_2_ and applying a stimulus from resting P_ET_CO_2_ to resting + 10 mmHg. It was counterintuitive that a P_ET_CO_2_ stressor of 34 mmHg to 44 mmHg in one person with resting P_ET_CO_2_ of 34 mmHg would be equivalent to one of 44 mmHg to 54 mmHg in another with resting P_ET_CO_2_ of 44 mmHg. However, over time we were convinced that this was indeed the case and changed to a stressor P_ET_CO_2_ spanning the range from baseline to baseline + 10 mmHg. Figure 4 is a vivid illustration of the wisdom of this approach. These findings are consistent with the idea that people normalize their resting vascular tone to their resting PCO_2_ which they retain for years as indicated by full metabolic compensations and are not at all committed to some arbitrary “normal” value in textbooks.

From these considerations, we have the fifth lesson:

5. For accuracy of CVR and comparability between subjects, the baseline for CVR measurement should be the resting P_ET_CO_2_.

### Effect of Blood Pressure on CVR

**Figure 5:**
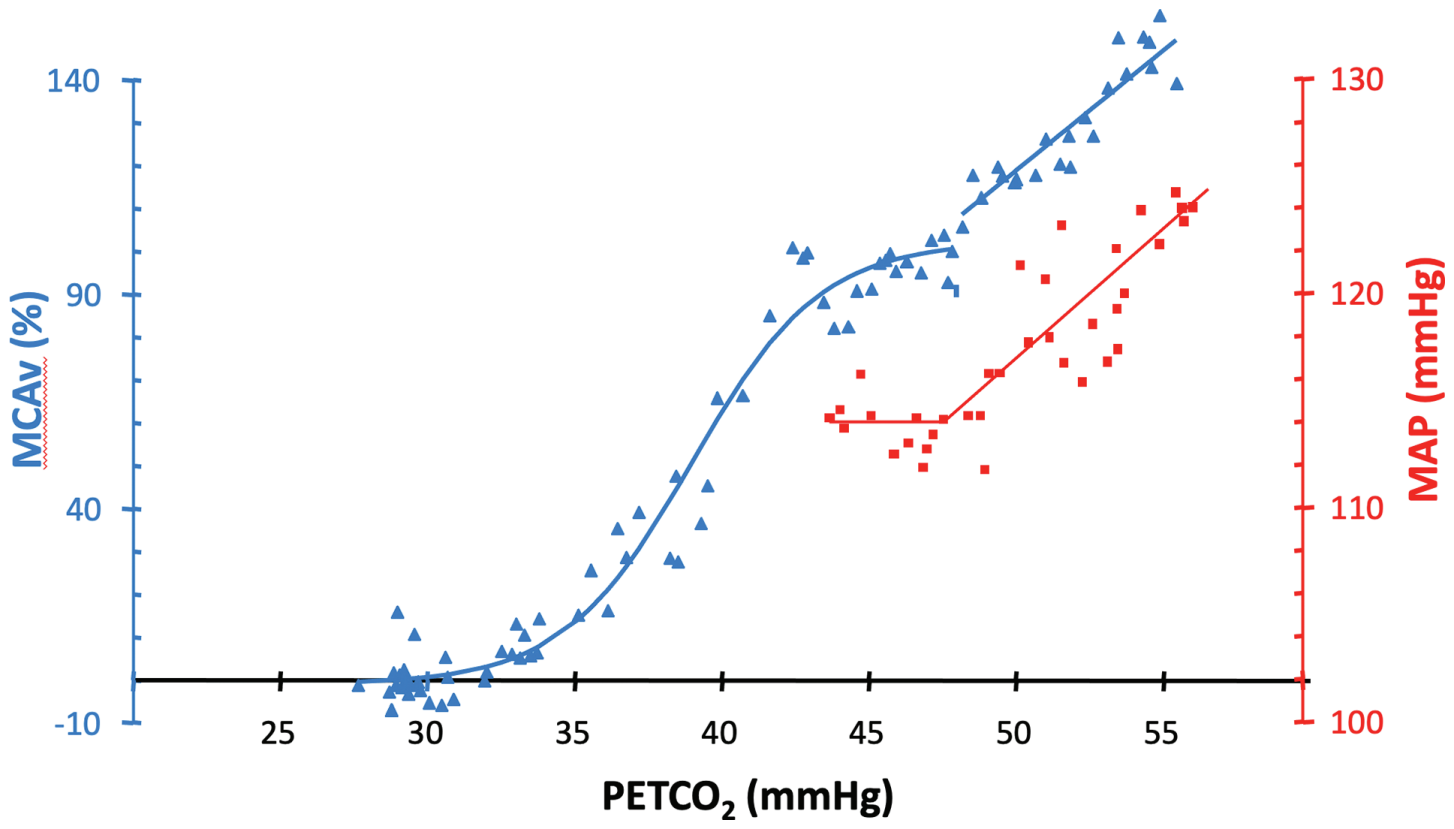
MAP (squares) and %MCAv (triangles) responses to increases in P_ET_CO_2_ in a healthy subject. In the presence of hypercapnia, an apparent “autoregulatory break-through” occurs in this subject at approximately 47 mmHg. Figure recreated and modified from Battisti-Charbonney et al. (2011).

The brain is protected from hypoperfusion resulting from reductions in perfusion pressure by reflex vasodilation, mostly of small arterioles (50–200 μm) downstream from pial vessels (~300 μm; Kontos, 1989), a response termed “autoregulation” (Kulik et al., 2008). Hypercapnia is distressing for some subjects, and a few may respond with increases in blood pressure. Hypercapnia dilates both the pial and penetrating arterioles, blunting the autoregulatory vasoconstriction restraining increases in CBF due to increases in blood pressure. In the presence of hypercapnia, an apparent “autoregulatory break-through” may occur, as depicted in Figure 5.

From these considerations, we have the sixth lesson:

6. Monitor blood pressure during assessment of CVR. Interpret flow results in the presence of hypercapnia and hypertension with caution.

### The Effect of PO_2_ on CVR

When administering a fixed FICO_2_ such as “carbogen” (5% CO_2_), whether the balance is composed of air or O_2_ is consequential. If the balance is O_2_, then compared to the balance being air, there will be a higher ventilatory response and, as a result, a smaller ΔPaCO_2_ (Prisman et al., 2007; Fisher, 2016) and therefore a smaller ΔCBF. Concurrent hypoxia to arterial saturations below 70% may also increase the CBF response to hypercapnia (Poulin et al., 1996; Ainslie and Burgess, 2008; Mardimae et al., 2012; Willie et al., 2012).

Accordingly, seventh and eighth lessons are:

7. With fixed FICO_2_, the minute ventilation, and therefore the PaCO_2_, depends on the inspired PO_2_ and ventilatory response, neither of which can be predicted or corrected *post hoc*. This confounder should be considered for all fixed FICO_2_ protocols.
8. A fixed inspired PO_2_ does not result in a fixed arterial PO_2_; the latter varies depending on the level of ventilation in a mechanism analogous to that of breathing a fixed FICO_2_. Such small changes in PO_2_ due to differences in ventilation have inconsequential changes for the CVR.

### Non-sigmoidal Flow Responses and Calculation of CVR

**Figure 6:**
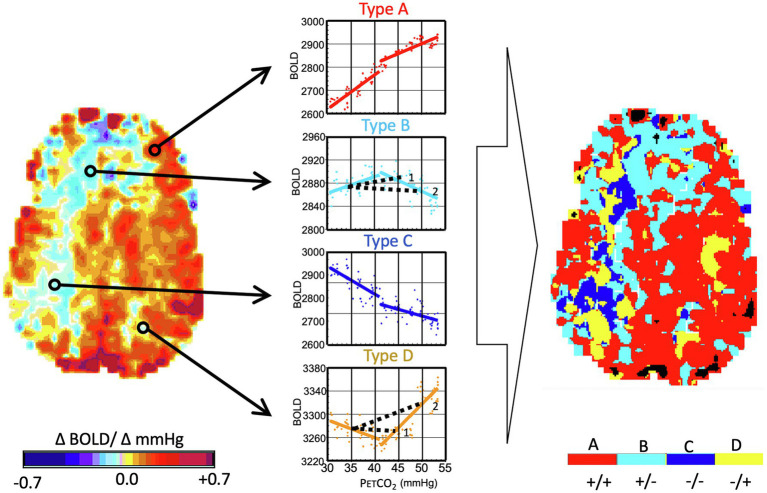
Analysis of flow signal (ΔBOLD) over a range of PaCO_2_ in the presence of cerebrovascular disease. Same subject as Figure 1. The PaCO_2_ was varied in a ramp paradigm (see Figure 3) from 10 mmHg below baseline to 15 mmHg above baseline. *Left Map:* The voxels are analyzed as CVR and color-coded as per the color scale. *Right Map:* This map uses the color scale to indicate the pattern of BOLD signal over the range of PaCO_2_. Types of response: **(A)** positive response with a sigmoidal shape; **(B)** initial positive response which then declines; **(C)** response which progressively declines; and **(D)** initial decline followed by progressive increase. In healthy people, robust response voxels overwhelmingly predominate, and the map is substantially red. Note the projection of CVR as calculated for a hypothetical two-point PCO_2_ stimulus of 45 mmHg and 50 mmHg for the voxels of type (B) and (D) (see 1 and 2). In the upper figure, a P_ET_CO_2_ stimulus of 45 mmHg results in a positive CVR but at a P_ET_CO_2_ of 50 mmHg, CVR of the same voxel is negative, indicating “steal.” In the lower figure, a P_ET_CO_2_ of 45 mmHg results in a negative CVR but a positive CVR at 50 mmHg. Importantly, the type map can define the regions with the greatest steal physiology, as indicated by dark blue. This can then be used as a voxel-wise ordering of severity of steal physiology. Modified from Duffin et al. (2017).

In our early investigations of the way in which CBF responds to PaCO_2_, we applied a gradual “ramp” increase in PaCO_2_ from resting baseline to baseline + 15 mmHg over 4 min (Sobczyk et al., 2014). The results made it clear that, in the presence of cerebrovascular disease, many vascular beds, some as large as an entire hemisphere, have complex flow responses that are decidedly not sigmoidal, nor could they be accurately summarized by a linear regression. We studied the voxel-by-voxel changes in signal over the range of P_ET_CO_2_ (Fisher et al., 2017) and were able to classify the P_ET_CO_2_ response patterns into four basic types: proportional to PaCO_2_, biphasic rise-fall, negatively proportional to PaCO_2_, and biphasic fall-rise (Figure 6).

By investigating the frequency and distribution of the various response patterns in healthy subjects and patients with cerebrovascular steno-occlusive disease, we found that the response types were not dispersed throughout the brain, but tended to cluster in large contiguous regions, strongly suggesting shared physiologic causes. Analyzing the biphasic curves as a linear regression can obscure important physiological information from the subsequently derived CVR maps (Fisher et al., 2017). Rather, multimodal analysis (transfer function analysis (Duffin et al., 2015) or vascular resistance analysis (Duffin et al., 2017, 2018) can be used to extract abundant additional nuanced information regarding vascular response functions.

From these investigations, we derived the additional lessons:

9. A ramp stimulus reveals the pattern of response to a range of PaCO_2_ values.
10. A ramp stimulus to a PaCO_2_ >10 mmHg above resting explores a higher range of vasodilatory reserve. This range of reserve would not be interrogated with a smaller stressor such as 5 mmHg.
11. A ramp stimulus generates the data in a form that can be analyzed for intrinsic vascular resistance (Duffin et al., 2017, 2018; McKetton et al., 2019).

## A New CVR Metric: Speed of Response

**Figure 7:**
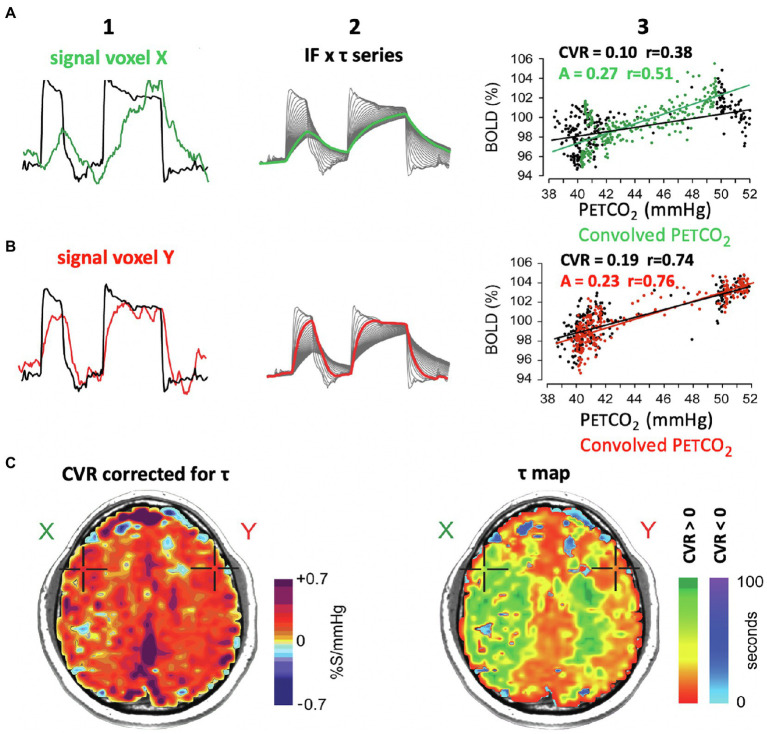
Effect of the speed of vascular response to a stimulus on the measure of CVR. **(A1**, **B1)**: P_ET_CO_2_ and BOLD signal for voxels shown in maps in row (C). **(A2**, **B2)**: multiple versions of P_ET_CO_2_ obtained by convolving the P_ET_CO_2_ with an exponential function of time constant *τ*. The heavy line is the one that best fits the signal shown in A1 and A2, and the associated τ characterizes the speed of vascular response (see Figure 1 in Poublanc et al., 2015). **(A3, B3)**: The black dots map the BOLD vs. P_ET_CO_2_, and the black line indicates the associated regression line. The green and red dots are BOLD signal graphed against *convolved* P_ET_CO_2_. The slopes of the green and red regression lines denote the amplitude of the signal corrected for its response time *τ*. (**C**) Right figure shows the distribution of *τ* in an axial slice. The slowed responses (indicated by the green coloration) would tend to reduce the CVR value calculated from actual P_ET_CO_2_ and increase the “steal” colors calculated by line of best fit alone (see images on left in Figure 4). **(C)**: Left figure. The CVR map shows a normal CVR after mathematically correcting for the slowed *τ*. This shows that abnormal CVR calculations without correcting for *τ* would be due to a slowed *τ*.

The calculation of CVR as the slope of a regression between PaCO_2_ and flow becomes progressively less representative of vascular reactivity as the correlation diminishes. However, another, less apparent factor also affects the quality of representation, and likely a reliable indicator of vascular health in its own right—duration of full evolution of the CVR response after stimulus onset, i.e., the “*speed of response*.” The effect of a slow response on the calculation of CVR is shown in Figure 7. The slow vascular response to a step change in P_ET_CO_2_ (Figure 7A1, green line) graphs a large range of delayed changes in BOLD signal at the 50-mmHg position on the abscissa (Figure 7A3, black dots) reducing the slope of the line of regression, compared to that of a rapid response (Figure 5 row B, red line, black dots). Clearly, a simple regression of all data points is not an accurate summary of the vascular response when the vascular response time to the stimulus is prolonged; a correction is therefore required.

### Mathematical Correction of CVR for the Speed of Response

In Figure 7 we show the mathematical approach of Poublanc et al. (2015) for correcting CVR for a slow response. This process entails assuming the response is a first-order exponential and obtaining an index of that delay in the form of τ, the time constant required to match the response to the exponential function (Figures 7A2,B2). Caveat: Heretofore, we have used the input function containing both the rising and declining PaCO_2_ in the calculation of τ in response to the step change in PaCO_2_. This method assumes *τ* is the same in both directions. Figures 2, 3 provide reason to challenge this assumption in future analysis. Further mathematical treatments are needed. Transfer function analysis can be used to identify phase, gain, and coherence metrics (Duffin et al., 2015). Sinusoidal stimuli may be used (Blockley et al., 2011), and the linear fit between the observed BOLD signal and the arrival-time adjusted P_ET_CO_2_ convolved with the hemodynamic response function (Yao et al., 2021).

### Physical Correction of CVR for the Speed of Response

**Figure 8:**
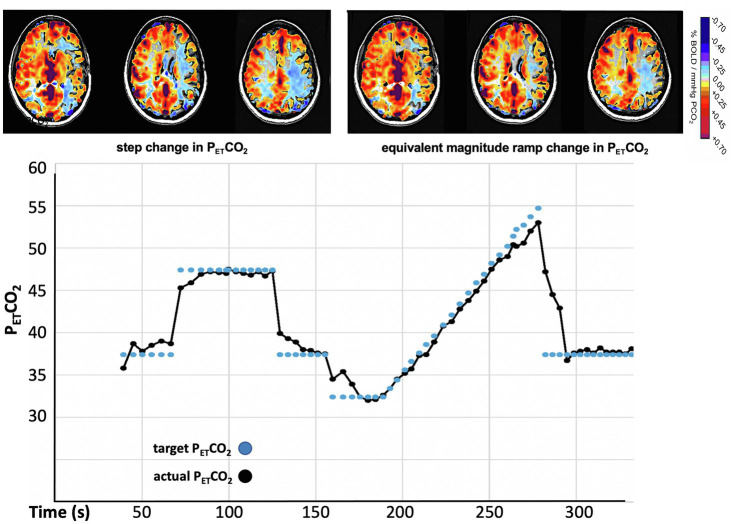
CVR measured with a step (“box car”) change and a ramp change of P_ET_CO_2_ in a patient with moyamoya disease. The calculation of CVR after the step change indicates extensive bilateral “steal.” The gradual ramp stimulus shows much reduced steal (less blue). These maps indicate that, if not accounted for, slowed response times contribute to the reduced values in the calculation of the CVR.

The amplitude of CVR can also be corrected for speed of response by changing the P_ET_CO_2_ vs. time profile of the stressor from a step to a ramp (Figure 8). In the case of a slowly rising stressor, the effect of slowed response times on the CVR is reduced, resulting in an accurate calculation of amplitude of CVR (Poublanc et al., 2015).

### Speed of Response as a Metric of Vascular Health

How quickly the vascular resistance in a voxel changes may be a metric of neurovascular health. We frequently observed a strong correlation between speed of response, CVR, and known vascular pathology. Our current impression is that of these three, the speed of response is the most sensitive to mapping the extent and grading the severity of pathology (Poublanc et al., 2015; Holmes et al., 2020), a conclusion also reached by others (van Niftrik et al., 2017). It is currently debated as to whether the *τ* slowing effect of vascular risk factors on parenchymal brain arteries can be separated from those of extra-parenchymal large vessel arterial steno-occlusive disease.

Measurement of the speed of response may be limited by the time taken for the stimulus to evolve to a steady state. Figures 7A2,B2 illustrates a range of the slowing of response to a square wave stimulus. If rather than a square wave, the PaCO_2_ rises to a maximum with a time constant of *τ*_stimulus_, the calculated *τ*_response_ cannot be less. With stressors such as fixed FICO_2_, it is not possible to measure any τ-_response_ less than the time constant of the lung wash-in to a new PCO_2._ In healthy young people, this is 20–30 s but is longer in older people and those with expiratory flow restrictions. Consequently, measuring the intrinsic speed of the vascular response requires a stressor with a very short *τ*-_stimulus_ approaching a square wave, substantially changing from baseline to target PaCO_2_ within one breath, as is possible with SGD (Fisher et al., 2016).

From these considerations, we have drawn three further lessons:

12. CVR has two independently measurable metrics: the *amplitude* of response and the *speed* of response.
13. The measure of the speed of vascular response to a stimulus requires a stimulus shorter than the fastest vascular response time constant to be measured.
14. To measure amplitude of change, speed of change can be accounted for mathematically or physically *via* a slow ramp or sinusoidal stimulus paradigms.

## Assessing Single Subjects/Patients: What is Normal?

**Figure 9:**
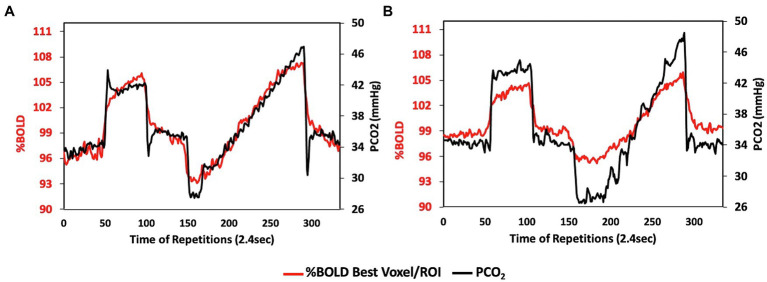
Reproducibility of the stimulus. Data from a 46-year-old female being followed for progressive narrowing of intracranial segments of both internal carotid and middle cerebral arteries. CVR scan in **(A)** was performed 2 years prior to CVR scan in **(B)** to assess the progress of hemodynamic compromise. Note the high reproducibility of the stimulus. This reproducibility enables the dampening flow response (red line) in **(B)** to be attributed to the vascular compromise.

**Figure 10:**
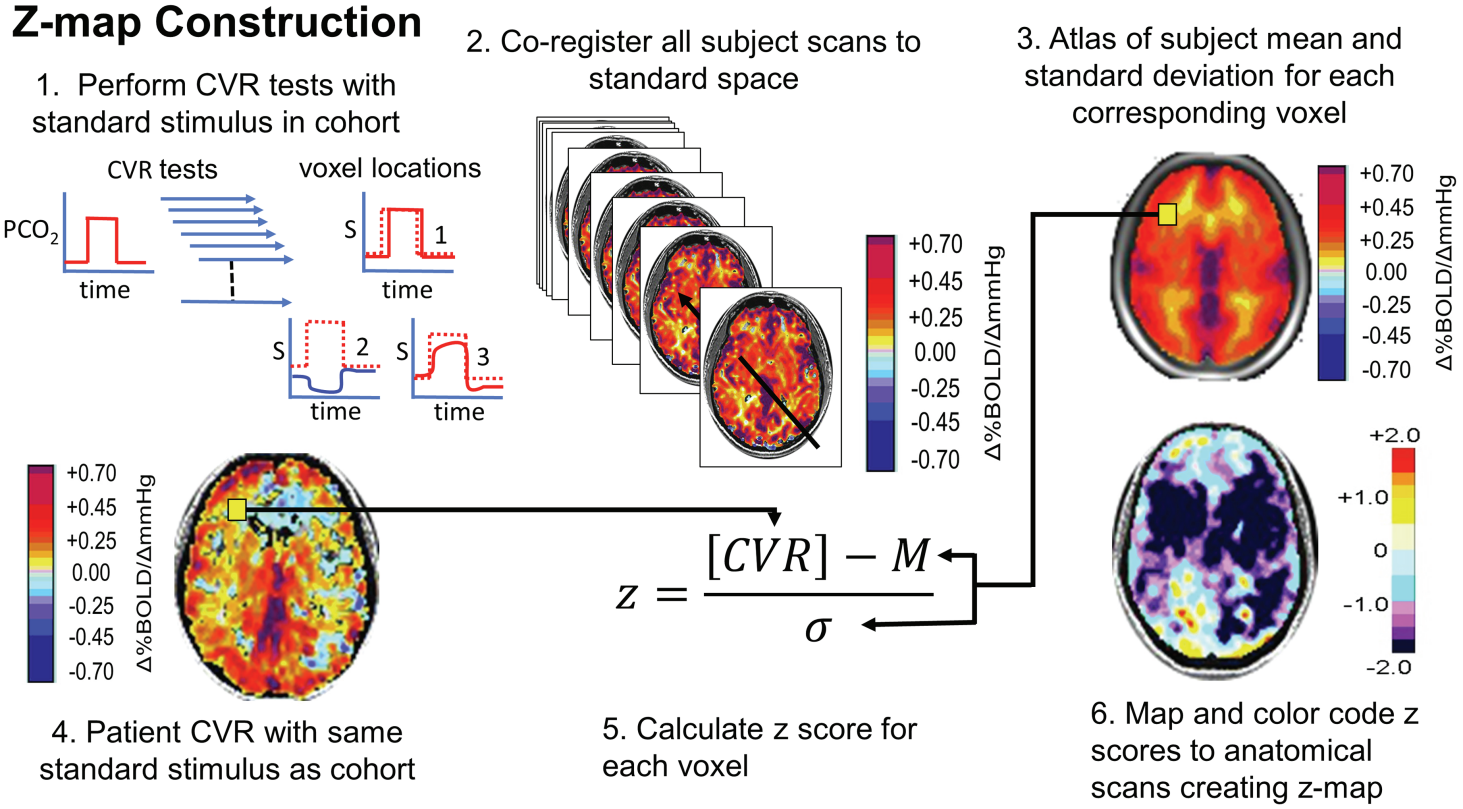
Normalizing the CVR score in terms of the normal range. An identical stimulus is applied to each subject in a cohort of healthy people. CVR is calculated and mapped to standard space. Mean (M) and standard deviation (*σ*) are calculated for each voxel. A patient is administered the same test. The patient CVR is assessed as z-scores, defined as shown in step 5. Z-scores can be color-coded and mapped over the anatomical scan to show the distribution of coherence to the mean. Figure from Fisher et al. (2018).

The repeatability of a test in one person over time (Figure 9) or between two people is the key to clinical application. Assuming two subjects receive identical stimuli and are scanned with the same scanner parameters, the CVR of a ROI, or even that of a single voxel, should be comparable after accounting for day-to-day variations in subject physiology and scanner instabilities. Administering the same stimulus and using the same scan parameters over a cohort of healthy subjects enable the generation of a map of voxel-wise normative CVR values (as mean and standard deviation) controlled for anatomical location, age, sex, or any other factor. Collectively, such normative data are referred to as a “CVR atlas” (Sobczyk et al., 2015; Figure 10).

Performing repeated CVR tests in a cohort enables the documentation of the normal range of voxel-wise test–test variability. These data can be used to assess whether repeated tests in a single subject differ over and above that due to extrinsic causes (e.g., stimulus delivery and imaging devices) of test–retest variability and therefore attributable to a disease process or surgical intervention. Such determinations are pertinent in clinical assessment and drawing conclusions from clinical data (Sobczyk et al., 2016).

From these considerations, we draw the final two lessons:

15. A standard stimulus and uniform scan parameters enable construction of an atlas of normal values and their standard deviation.
16. A merged group atlas of normative CVR metrics enables scoring a single subject/patient in terms of standard deviation differences from normal responses, thus providing clinical utility important for judging the impact of steno-occlusive disease in that individual.

## Conclusion

Standardization and reproducibility of the CO_2_ stressor are fundamental to advance the use of CVR for understanding cerebral vascular physiology and pathophysiology and translating the science to the bedside.

We present 16 lessons (limitations, optimizations, and interpretations) acquired from two decades using CVR as a cerebrovascular stress test in about 2,000 patients with cerebrovascular disease, as well as several healthy subjects. We investigated the method of action, and thereby the optimization and limitations of the stressor, identified suitable outcome variables, and divined the sometimes very obscure physiology at which they hinted. Many, but not all, of these have been presented in one way or another in our peer-reviewed publications over the years and various journals but we believe will benefit from collation into one document. The sum of this work may lead to the development of a quantitative repeatable standardized CVR test for both clinical and research purposes. In patients, it can be used to detect and quantify hemodynamic insufficiency, follow the natural history of the disease in individual patients, and assess the response to interventions. For research, the ability to apply standardized CVR methodology for data acquisition and quantitation is critical in any prospective clinical trial that assesses the natural history of disease and its management.

## Data Availability Statement

The data analyzed in this study is subject to the following licenses/restrictions: Anonymized data will be shared by request from any qualified investigator for purposes such as replicating procedures and results presented in the article provided that data transfer is in agreement with the University Health Network and Health Canada legislation on the general data protection regulation. Requests to access these datasets should be directed to OS, olivia.sobczyk@uhn.ca

## Ethics Statement

The studies involving human participants were reviewed and approved by University Health Network, Toronto, Canada. The patients/participants provided their written informed consent to participate in the studies. Written informed consent was obtained from the individual(s) for the publication of any potentially identifiable images or data included in this article.

## Author Contributions

OS, JF, LV, JD, JAF and DJM drafted the manuscript. All authors participated in the feedback and writing process following the initial drafting of the manuscript.

### Conflict of Interest

JAF and DJM contributed to the development of the automated end-tidal targeting device, RespirAct^™^ (Thornhill Research Inc., (TRI)) used in the figure studies mentioned and have equity in the company.RespirAct^™^ is not currently a commercial product but is made available for REB-approved research. OS and JD received salary support from TRI. TRI provided no other support for the study.

The remaining authors declare that the research was conducted in the absence of any commercial or financial relationships that could be construed as a potential conflict of interest.

The reviewer RH declared a past collaboration with one of the authors JAF to the handling editor.

## Abbreviations

BOLD: blood oxygen-level dependent;
CBF: cerebral blood flow;
CVR: cerebrovascular reactivity;
FICO_2_: fractional concentration of inspired carbon dioxide;
P_ET_CO_2_: end-tidal partial pressure of carbon dioxide;
MAP: mean arterial pressure;
MCA: middle cerebral artery;
MCAv: middle cerebral artery velocity;
MRI: magnetic resonance imaging;
PaCO_2_: arterial partial pressure of carbon dioxide;
ROI: region of interest;
SGD: sequential gas delivery.
